# Draft Genome Sequence of *Alternaria alternata* Isolated from Onion Leaves in South Africa

**DOI:** 10.1128/genomeA.01022-16

**Published:** 2016-09-22

**Authors:** Wubetu Bihon, Michele Cloete, Abe Shegro Gerrano, Dean Oelofse, Patrick Adebola

**Affiliations:** a World Vegetable Center–West and Central Africa, Samanko Research Station, Bamako, Mali; b Department of Microbiology and Plant Pathology, Forestry and Agricultural Biotechnology Institute (FABI), University of Pretoria, Pretoria, South Africa; c Vegetable and Ornamental Plants–Agricultural Research Council, Pretoria, South Africa; d Africa Rice Center (AfricaRice), c/o Central Agricultural Research Institute (CARI), Suakoko, Bong County, Monrovia, Liberia

## Abstract

*Alternaria alternata* (Fr.) Keissler strain PPRI 21032 was isolated from onion leaves collected in Roodeplaat, Pretoria, South Africa. The whole genome of this strain was sequenced and produced a total of 33.12 Mb with a GC content of 50.9%. The whole genome comprises 11,701 predicted coding sequences.

## GENOME ANNOUNCEMENT

*Alternaria* is a common fungal genus that includes saprobic, endophytic, and pathogenic species. Pathogenic *Alternaria* spp. cause major losses of many crops worldwide (1, 2). *A. alternata* is the most ubiquitous species in the genus and infects more than one hundred cultivated and uncultivated plants (3). It produces more than 60 secondary metabolites, including mycotoxins (4). *A. alternata* is synonymized to 35 morphotypes that cannot be distinguished based on multigene phylogeny (3). *A. alternata* was isolated from onion leaves in South Africa (5), and we report here the whole genome sequence of strain PPRI 21032.

*A. alternata* strain PPRI 21032 was grown on malt extract agar, and genomic DNA was extracted using a DNeasy plant minikit (Qiagen, Aarhus, Denmark). Whole-genome sequencing was performed using an Illumina MiSeq platform with pair-end libraries and insert sizes of 250 bp at the Agricultural Research Council, Biotechnology Sequencing Platform, Pretoria, South Africa.

Sequence reads were subjected to quality filtering and trimming, and *de novo* assembly was performed using CLC Genomics Workbench version 8.0.1 (CLCBio, Aarhus, Denmark). Quality of the assembled data was assessed based on the *N*_50_ value and the number of contigs. Quality and completeness of the assembled genome were estimated using benchmarking universal single-copy orthologs (BUSCO) software with a fungal data set on all contigs greater or equal to 500 bases (6). Putative open reading frames were predicted using the online version of the *de novo* prediction software AUGUSTUS (7) based on the gene models for *Aspergillus oryzae*.

The *de novo* assembly generated a total of 552 contigs, with the largest contig containing 1,086,627 bases. The assembled sequence has an *N*_50_ of 229 kb with an average GC content of 50.9%. The whole draft genome of *A. alternata*, isolated from onion leaves, has an estimated size of 33.12 Mb, comprising 11,701 predicted genes, based on the AUGUSTUS analysis. The BUSCO analysis of the completeness of this genome sequence predicted that 97% was complete. Out of 1,438 total BUSCO groups searched, the assembly contained 1,398 complete single-copy BUSCOs, 126 complete duplicated BUSCOs, 15 fragmented BUSCOs, and 25 missing BUSCO orthologs. The total genome size and the number of predicted genes are similar to other *A. alternata* genomes **(**2, 8). This is a draft genome report, but it will provide useful information for genome comparisons within the species *A. alternata* and the whole *Alternaria* genus, as well as for finding innovative techniques to study the biology of this and other related microorganisms.

### Accession number(s).

This whole-genome shotgun project has been deposited at DDBJ/ENA/GenBank under the accession number LSHC00000000. The version described in this paper is the first version, LSHC01000000.
